# Spatial organization-dependent EphA2 transcriptional responses revealed by ligand nanocalipers

**DOI:** 10.1093/nar/gkaa274

**Published:** 2020-04-30

**Authors:** Toon Verheyen, Trixy Fang, Dominik Lindenhofer, Yang Wang, Karen Akopyan, Arne Lindqvist, Björn Högberg, Ana I Teixeira

**Affiliations:** 1 Department of Medical Biochemistry and Biophysics, Karolinska Institute, Stockholm 17165, Sweden; 2 Department of Cell and Molecular Biology, Karolinska Institute, Stockholm 17165, Sweden

## Abstract

Ligand binding induces extensive spatial reorganization and clustering of the EphA2 receptor at the cell membrane. It has previously been shown that the nanoscale spatial distribution of ligands modulates EphA2 receptor reorganization, activation and the invasive properties of cancer cells. However, intracellular signaling downstream of EphA2 receptor activation by nanoscale spatially distributed ligands has not been elucidated. Here, we used DNA origami nanostructures to control the positions of ephrin-A5 ligands at the nanoscale and investigated EphA2 activation and transcriptional responses following ligand binding. Using RNA-seq, we determined the transcriptional profiles of human glioblastoma cells treated with DNA nanocalipers presenting a single ephrin-A5 dimer or two dimers spaced 14, 40 or 100 nm apart. These cells displayed divergent transcriptional responses to the differing ephrin-A5 nano-organization. Specifically, ephrin-A5 dimers spaced 40 or 100 nm apart showed the highest levels of differential expressed genes compared to treatment with nanocalipers that do not present ephrin-A5. These findings show that the nanoscale organization of ephrin-A5 modulates transcriptional responses to EphA2 activation.

## INTRODUCTION

Erythropoietin-producing hepatoma (Eph) receptors are the largest class of receptor tyrosine kinases, comprising of 14 members classified as EphA and EphB in humans (1). Eph signaling is typically initiated by cell-cell interactions with transmembrane ephrins, which can generate uni- or bi-directional signaling (2). These juxtacrine cues regulate fundamental cellular processes like cell proliferation, differentiation, adhesion, migration and tissue patterning and morphogenesis in embryonic development and tissue homeostasis (3–7). Therefore, dysregulated Eph signaling is associated with cancer development, showing tumor-suppressive or oncogenic properties (8–12). To generate robust Eph signaling, there has been ample evidence showing that ephrins must be presented as multimers to induce kinase activation and receptor clustering (13–17). Eph activation is commonly performed *in vitro* by antibody-clustered ephrins and recombinant ephrin-Fc fusion proteins (14,18) as they induce tyrosine phosphorylation at a much higher potency than ephrin monomers (13). Recently, engineered peptide derivatives were shown to stabilize EphA2 dimers, promote receptor oligomerization and differentially modulate downstream phosphorylation events (17,19,20). This evidence reflects the importance of receptor dimerization and oligomerization for Eph signal propagation.

The spatial organization of Eph receptors has been increasingly shown to be important in regulating receptor activity (21,22). For example, restriction of lateral movement of EphA2 receptors with chromium barriers disrupted recruitment of downstream Eph effectors, altered cytoskeleton morphology (23) and impeded receptor endocytosis in MDA-MB-231 cells (24). In addition, modulating the nanoscale spacing of ephrin-A5 dimers to direct EphA2 receptors at defined positions using DNA origami nanostructures tuned receptor phosphorylation levels in human breast cancer MDA-MB-231 cells, resulting in reduced cell invasion (25). However, the impact of nanoscale spatial organization of ephrins on downstream Eph receptor signaling and overall transcriptome is unknown. Moreover, very little is known about Eph receptor signaling in glioblastoma (GBM) despite that EphA2 overexpression drives cell proliferation via MEK/ERK in GBM (26–28) and cell invasion via AKT signaling in glioma stem cells (29).

Here, we use ephrin-A5-decorated DNA origami nanostructures (ephrin-A5 DNA nanocalipers) to form ligand nanoassemblies with well-defined spatial organizations, which tune EphA2 phosphorylation in human GBM and breast cancer cells and also downstream signaling in GBM.

## MATERIALS AND METHODS

### Ephrin-A5 conjugation

Recombinant ephrin-A5-Fc-Chimera (R&D Systems, Cat. No. 374-EA-200) was conjugated to an azide-oligo via its His-tag utilizing the *bis*-sulfone-PEG_4_-dibenzocyclooctyne(DBCO) compound (Kerafast) as previously described (30). In brief, *bis*-sulfone-PEG_4_-DBCO was resuspended in *N*,*N*-dimethylformamide at 10 mM concentration. It was then diluted to 5 mM in 1× PBS, pH 6.3 and incubated for 1 h at ambient room temperature. The ephrin-A5-Fc chimera protein was resuspended in 1× PBS, pH 6.3 at a concentration of 0.2 mg/ml. The *bis*-sulfone-PEG_4_-DBCO and ephrin-A5 were mixed together at a molar ratio of 20:1 and incubated at ambient room temperature for 4 h. A Zeba Spin Desalting Column (7K MWCO, 0.5 ml) (ThermoFisher Scientific) was used to remove the unconjugated compound and buffer exchange the protein mixture in 1× PBS, pH 7.4. The azide-oligo (5′ CTCTCCTTCTTCCCTTTCTTT/3AzideN/ 3′) was added to the buffer-exchanged protein mixture at a molar ratio of 3:1 (azide-oligo:DBCO moiety). The DBCO incorporation was determined by measuring its absorbance at 309 nm. The mixture was then incubated at ambient room temperature overnight. The conjugates were then purified using an Amicon Ultra-0.5 centrifugal filter unit with Ultracel-50 membrane (Merck Millipore) coated with 5% Pluronic-F127 (w/v) (ThermoFisher Scientific). The purification was performed by centrifuging the sample at 14 000 × *g* for 1 min, resuspending the concentrate in 1× PBS, pH 7.4 and concentrating the sample again, repeating this 4–7 times. The column was then inverted into a clean eppendorf tube and centrifuged for 2 min at 1000 × *g*. The concentration of the purified conjugate was determined using the Micro BCA Protein Assay kit (ThermoFisher Scientific) following manufacturer's guidelines.

Validation of the protein-oligo conjugate and its purity were performed by hybridizing 4 μM of complementary fluorescently-tagged oligo (5′ AAAGAAAGGGAAGAAGGAGAG/3AlexaFluor647N/ 3′) to 2 μM protein-oligo conjugate for 1 h at 37°C. This mixture was then run on a native 4–20% Mini-Protean TGX Precast gel (Bio-Rad) in 1× Tris-glycine buffer at 200 V for 34 min and imaged with ImageQuant LAS 4010 system (GE Healthcare) with Cy5 transillumination. Proteins were detected using the Pierce Silver Stain kit (ThermoFisher Scientific).

### Ephrin-A5 nanocaliper production

The ephrin-A5 DNA nanocalipers were produced as previously published with minor alterations (25). The p7560 single-stranded scaffold DNA from M13mp18 phage was purchased from Tilibit. After folding of the DNA nanocalipers, ephrin-A5-oligo conjugates were added at a molar excess of 5-fold (1 site) or 10-fold (2 sites) to the nanocalipers. Excess ephrin-A5 conjugates were then removed by passing the ephrin-A5 nanocalipers consecutively through two 0.8 ml centrifuge columns, each containing 400 μl compacted Sepharose 6B resin (Sigma-Aldrich). After purification, the ephrin-A5 nanocalipers were concentrated using a 10-kDa MWCO 0.5 ml Amicon Centrifugal Filter (Merck Millipore). The structures were then run on a 2% agarose gel stained with 0.5 mg/ml ethidium bromide (Sigma-Aldrich) to determine both the quality and concentration of the ephrin-A5 nanocalipers.

Using ImageJ (NIH), the migration distances of the ephrin-A5 nanocalipers in between agarose gels were determined by drawing a line plot across each band and extracting the grey values and distance values across it. The size of the line plot was determined by the distance between the 700 and 3000 bp bands of the 1 kb plus DNA Ladder (ThermoFisher Scientific) in each gel. The maximum grey value of each line plot was taken to be the gel band of the ephrin-A5 nanocaliper and its corresponding distance value was normalized by the distance value obtained from the NC-Empty maximum grey value. The values were then plotted using the *R* package ggplot2 (31).

### Cell culture

Patient-derived U3013 human glioblastoma (GBM) cell line was provided by the Human Glioblastoma Cell Culture resource (HGCC www.hgcc.se) at Uppsala University (32). Briefly, T25 flasks were coated with 10 μg/ml of poly-l-ornithine (Sigma-Aldrich) for 3 h at room temperature and then 10 μg/ml laminin (Sigma-Aldrich) for 30 min at 37°C, 5% CO_2_. After incubation, some laminin was retained for better cell attachment. U3013 cells were cultured in poly-ornithine/laminin-coated flasks in GBM media (1:1 DMEM/F-12 GlutaMAX and Neurobasal medium (ThermoFisher Scientific) with the addition of 1.67 nM EGF (R&D Systems), 0.56 nM bFGF (ThermoFisher Scientific), 2% B27 (ThermoFisher Scientific) and 1% N2 Supplement (ThermoFisher Scientific)) supplemented with 1% penicillin/streptomycin. Human breast cancer MDA-MB-231 cell line (ATCC) were cultured in DMEM (Gibco) supplemented with 10% fetal bovine serum and 1% penicillin/streptomycin.

### *In situ* proximity ligation assay (PLA)

U3013 cells were seeded on poly-ornithine/laminin-coated glass coverslips in a 24-well plate at 10 000 cells per well for 24 h in GBM media before nanocaliper stimulation. MDA-MB-231 cells were seeded similarly on ethanol-cleaned coverslips and subjected to 24 h of reduced serum conditions (DMEM supplemented with 0.5% fetal bovine serum and 1% penicillin/streptomycin). The cells were stimulated with the following nanocaliper conditions; 20 nM NC-empty, 20 nM NC-0, 10 nM NC-14, 10 nM NC-40 and 10 nM NC-100 in 1× PBS with 13 mM MgCl_2_. Briefly, the coverslip upon which the cells were seeded on was inverted onto a drop of ephrin-A5 nanocaliper solution on parafilm, with the cell-side contacting the drop. For U3013, 1.67 nM EGF and 0.56 nM bFGF were supplemented into the ephrin-A5 nanocaliper mix. The cells were stimulated for 15 and 30 min at 37°C, 5% CO_2_ and then washed with ice-cold 1× PBS. The cells were fixed in 10% formalin for 20 min and washed three times in 1× PBS for 5 min. The PLA procedure was then carried out according to manufacturer's guidelines as previously described with minor alterations (25). For the labeling of EphA2 tyrosine phosphorylation, the following antibodies were used: anti-phosphotyrosine (1:727.27, Abcam, cat. no. ab9319) and anti-EphA2 (Clone D7, 1:888.8, Millipore, cat.no. 05-480). Images of individual cells were acquired using a Zeiss AxioImager M2 at 1936px × 1460px with 40 magnification in a *z*-stack configuration in ‘centre-mode’ (10–20 stacks, 0.5 μm step size). The number of biological repeats were: *n =* 3–7 for IgG, *n =* 3–7 for IgG-clustered Ephrin-A5, *n* = 7 for NC-Empty, *n* = 5–6 for NC-0, *n =* 3–5 for NC-14, *n* = 3–6 for NC-40 and *n* = 4–6 for NC-100. The PLA signals were quantified based on DAPI and Phalloidin signals using a custom written plugin, jPLA for ImageJ (33) with 10–20 cells per condition.

### RNA-seq library preparation and sequencing

The RNA-seq experiment was based on the Smart-Seq2 protocol as described previously (34). In brief, 4000 cells were plated per well in a 96-well plate. The cells were treated with either 20 μg/ml AffiniPure Goat Anti-Human IgG, Fcγ Fragment Specific (Jackson Immunoresearch, 109-005-008) or IgG-clustered ephrin-A5 (pre-clustered with IgG at a mass ratio of 1:10 for 15 min at room temperature) or a specific ephrin-A5 nanocaliper in a total volume of 50 μl for 30 min at 37°C, 5% CO_2_. This was performed in duplicates which were pooled together after cell lysis. 5 μl of cell lysate was used for reverse transcription and PCR preamplification. These steps were performed at 20 and 50 μl respectively to prevent any inhibitory effects from occurring. For the tagmentation step, 200 pg of cDNA was used for input. The final libraries were prepared utilizing paired-end sequencing with the Nextera XT library prep kit (Illumina). The IgG-clustered ephrin-A5 RNA-seq was performed on the HiSeq2500 platform with a 2 × 126 setup using ‘HiSeq SBS Kit v4’ chemistry. The ephrin-A5 DNA nanocaliper RNA-seq was performed on the NovaSeq 6000 platform in aS1 Flow cell with a 2 × 51 setup.

### RNA-seq DE gene calling

The raw reads were processed and trimmed using Trimmomatic (v. 0.35) (35). The trimmed reads were then aligned to the Ensembl Human Genome GRCh37 and ERCC (for the IgG-clustered Ephrin-A5 data sets) using STAR (v. 2.2.1) (35,36). Count tables were then generated using HTSeq (v. 0.5.4p3) and differential expression was then carried out using *R* package DESeq (v. 1.24.0) according to its user guide (37,38). For further analysis of the DE genes obtained from the DESeq2 package, only genes with an adjusted *P*-value of 0.05 or above were included in the MDA-MB-231 and U3013 IgG-clustered ephrin-A5 data sets. For the U3013 cell line stimulated by the ephrin-A5 nanocalipers, only DE genes with an adjusted *P*-value of 0.1 or lower were included for downstream analysis.

Before determination of DE genes in the ephrin-A5 nanocaliper RNA-seq data analysis, genes that had an average RPKM value <1 were removed from the count tables. The DEseq2 analysis was then carried out using these count tables. The RNA-seq data sets were visualized using the Circos package (version 069.6) was used to create the Circos plots (39).

### Correlation analysis

The correlation analysis on whole data sets was carried out using the R package CORRR (v. 0.3.2) using default settings. Only genes that were detected in all 4 data sets, regardless of significance, were included in the analysis. For significant DE genes that were found to overlap between data sets, the genes and fold changes were extracted and a correlation coefficient was calculated using R and plotted accordingly. Proportional Venn diagrams for the gene overlaps between data sets were generated and adapted from BioVenn (40).

### Gene set enrichment analysis (GSEA)

The GSEA database from the Broad Institute was used to investigate gene set enrichment (41). Curated gene sets were used at a significance of FDR below 0.05 with default settings. Genes associated with an enriched pathway were discarded if they are present in another more significantly enriched gene set.

### Hypergeometric probability

KEGG and Reactome gene data sets were obtained from the GSEA database and hypergeometric probabilities were calculated against the nanocaliper RNAseq data sets to determine the significance of the overlap between the data sets (41). Only significantly enriched gene data sets (*P* < 0.05) were taken for further analysis. A few significant data sets were selected based on the relevance to the EphA2 signaling pathway and visualized using R.

### Network analysis

The network analysis was performed using a web-based version of GeneMANIA (42). The KEGG Spliceosome gene list was extracted from the GSEA database and used to interrogate the ephrin-A5 nanocaliper data sets (41,43). The overlapping genes from each data set was combined and used as input into the GeneMANIA server using default settings. The overlapping genes in each data set was then overlaid with its corresponding log_2_(FC) onto the obtained network.

## RESULTS

### Production of ephrin-A5 nanocalipers

**Figure 1.**
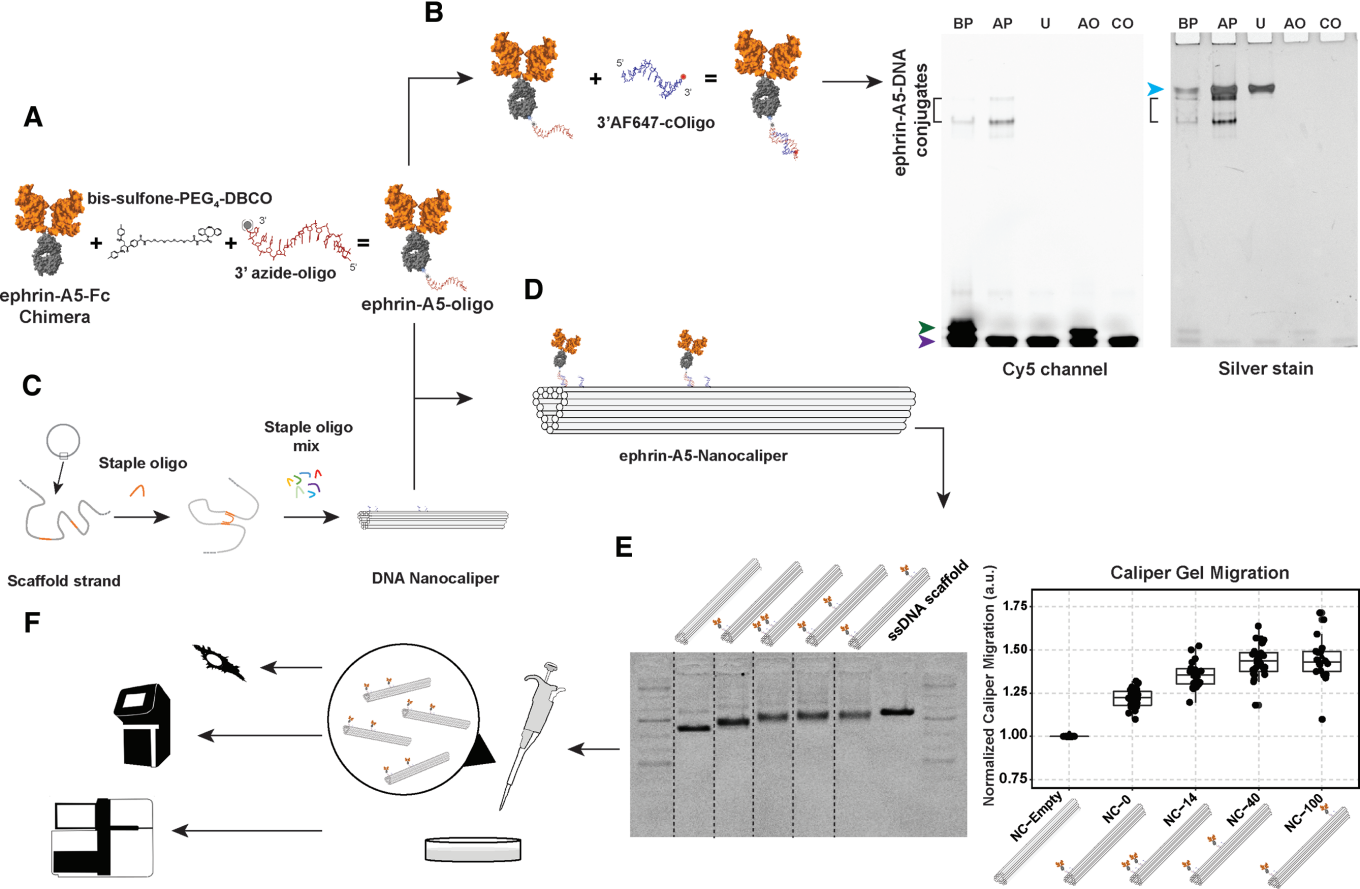
Ephrin-A5 nanocaliper production workflow. (**A**) Schematic representation of the conjugation of ephrin-A5-Fc-His to an azide-oligo using *bis*-sulfone-PEG_4_-DBCO as a crosslinker. (**B**) Native-PAGE of ephrin-A5-oligo conjugates hybridized to complementary Alexa Fluor 647-labelled oligos, visualized by fluorescence imaging followed by silver staining. Bands corresponding to ephrin-A5-oligo conjugates (black bracket) before (BP) and after purification (AP) were visualized by fluorescence imaging (Cy5 channel) and by silver staining. Unconjugated ephrin-A5 (U) and azide-oligos (AO) mixed with Alexa Fluor 647-oligos were used as controls to check for presence of unconjugated protein (cyan arrowhead) and free azide-oligos (green arrowhead) respectively. Another control containing only the Alexa Fluor 647-oligo (CO) was run to check for free fluorophore-oligos (purple arrowhead). (**C**) Folding of single-stranded circular phage DNA (scaffold) with short ssDNA oligos (staples) designed to complementarily bind to different regions of scaffold to create the DNA origami nanocaliper structure. (**D**) An example of a DNA nanocaliper functionalized with ephrin-A5-oligo conjugates spaced 42.9 nm apart. (**E**) Nanocalipers not functionalized with protein (NC-empty) as well as nanocalipers functionalized with ephrin-A5 at different positions (NC-0, NC-14, NC-40 and NC-100), p7560 scaffold DNA (ssDNA scaffold) and 1 kb Plus DNA ladder were analyzed using a 2% agarose gel stained with ethidium bromide (left). The gel image is a composite of different lanes within the same gel, with the dotted lines delineating each part of the composition. Gel migration distances of different ephrin-A5 nanocalipers normalized to NC-empty (right). The number of ephrin-A5 dimers attached to the nanocalipers was consistent with the step-wise migration shift observed (*n =* 32 (NC-empty), *n = 29* (NC-0), *n =* 18 (NC-14), *n =* 28 (NC-40), *n* = 18 (NC-100)). (**F**). Cells were stimulated by the addition of ephrin-A5 nanocalipers and analyzed with PLA, qRT-PCR and RNA-seq.

We have previously shown that ligand nanocalipers can be used to control the nanoscale spatial distribution of proteins (25). In particular, DNA oligos were conjugated to exposed lysines in ephrin-A5-Fc ligands using HyNic-4FB chemistry and hybridized to complementary oligos that protruded out of DNA nanotubes at defined positions, thereby creating ligand nano-patterns. Here, we report an improved production pipeline of ephrin-A5 nanocalipers that relies on poly-histidine tag (His-tag)-targeted conjugation of DNA oligos to ephrin-A5-Fc proteins. Other DNA conjugation strategies to proteins with Fc-domains involve protein G adaptors (44) or Fc-binding peptide-directed labeling (45). These methods result in site-specific conjugation of oligos to proteins, thereby avoiding potential loss of protein activity stemming from random oligo labeling of lysine residues (25). In our approach, we adapted a method developed for His-tag-specific PEGylation of proteins (30). First, the ephrin-A5-Fc-His protein was reacted with a *bis*-sulfone-PEG_4_-DBCO heterobifunctional crosslinker, leading to the formation of a stable covalent bond between the *bis*-sulfone moiety and the imidazole rings of the His-tag by *bis*-alkylation. After which, an azide-oligo was reacted with the DBCO group in a copper-free click chemistry reaction (Figure 1A). We confirmed the presence of ephrin-A5-Fc-oligo conjugates by hybridization of complementary fluorescently labeled oligos and then visualization on native-PAGE (Figure 1B). We observed two bands in the Cy5 fluorescence channel that were also visible after silver staining, indicating the formation of two ephrin-A5-Fc-oligo conjugate products that differ by the number of oligos attached. Additionally, a band that migrated more slowly than the conjugate bands was revealed by silver staining (cyan arrowhead), corresponding to unconjugated ephrin-A5-Fc-His homodimer. Furthermore, reducing SDS-PAGE showed a single conjugate band of approximately 58 kDa as well as a band corresponding to unconjugated ephrin-A5-Fc-His monomer (Supplementary Figure S1). Together, these results suggest that ephrin-A5-Fc-His homodimers were conjugated with one or two oligos, corresponding to the upper and lower conjugate bands, respectively, observed by native PAGE (Figure 1B). The fluorescence native-PAGE readout also detected any presence of unbound azide-oligos, which may interfere with the hybridization efficiency of the conjugates to the nanocalipers.

DNA nanocalipers were folded according to the pipetting scheme outlined in Supplementary Figure S2, using ‘staple’ DNA oligos (Supplementary Table S1), and then functionalized with purified ephrin-A5-conjugates via hybridization of the oligos present on the conjugates to the complementary protruding oligos in the nanocalipers (Figure 1C and D). Using this method, we produced a set of nanocalipers designed to present two ephrin-A5-Fc dimers spaced by design 14.3 nm (NC-14), 42.9 nm (NC-40) and 101.1 nm (NC-100) apart. We also produced nanocalipers with one ephrin-A5-Fc dimer (NC-0) and without any ephrin-A5-Fc dimers (NC-empty) as controls. To verify the functionalization of nanocalipers with ephrin-A5-oligo conjugates, we performed agarose gel electrophoresis (Figure 1E). The gel migration of NC-empty, NC-0 and NC-14 followed a distinct step pattern that confirmed successful functionalization as per design, with slower migration of nanocalipers with two ephrin-A5 dimers (NC-14, NC-40 and NC-100) as compared to nanocalipers with one dimer (NC-0) and without any dimers (NC-empty). We further verified the binding capacity of the ephrin-A5 nanocalipers to EphA2 receptor using an agarose gel shift binding assay (Supplementary Figure S3). We observed band shifts in ephrin-A5-DNA nanocalipers mixed with EphA2 receptors, which confirmed receptor binding to the ligands. Overall, the conjugation pipeline can be applied to produce nanocalipers functionalized with any protein of interest that contains a His-tag.

### Ephrin-A5 nanocalipers modulate EphA2 receptor phosphorylation in MDA-MB-231 and U3013 cell lines

To investigate the effect of nanoscale spatial distribution of ephrin-A5 on EphA2 activation, we studied EphA2 tyrosine phosphorylation using *in situ* proximity ligation assay (PLA) in MDA-MB-231 and U3013 cells stimulated with ephrin-A5 nanocalipers. We established a workflow for accurate quantification of EphA2 phosphorylation events with a custom-built ImageJ macro (Supplementary Figure S4). The cells were stimulated for 15 and 30 min, as levels of EphA2 phosphorylation activated by IgG-clustered ephrin-A5 peaked at these timings (Supplementary Figure S5).

**Figure 2.**
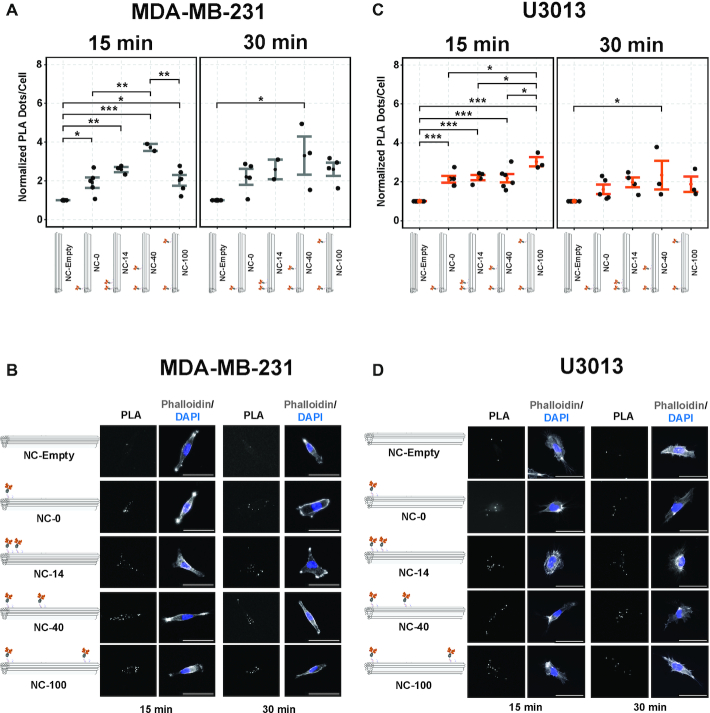
EphA2 receptor activation by ephrin-A5 nanocalipers in MDA-MB-231 and U3013 cell lines. (**A**) Quantification of EphA2 phosphorylation (pTyr) in MDA-MB-231 cells and (**C**) U3013 cells after 15- or 30-min stimulation with NC-empty, NC-0, NC-14, NC-40 and NC-100. *P* values indicated as * <0.05, ** <0.01, ***<0.001. Significance was determined by performing one-way ANOVA followed by Tukey HSD test. Error bars represent the standard error of the mean. Outliers were removed based on violations of normality and homogeneity of variances assumptions of ANOVA as determined by residuals analysis. Each dot represents the mean of each independent experiment containing 10–20 cells. (**B**) Representative PLA (EphA2-pTyr), DAPI (nucleus) and phalloidin (actin) microscope images of MDA-MB-231 cells and (**D**) U3013 cells. Scale bar denotes 50 μm.

NC-0, NC-14, NC-40 and NC-100 induced EphA2 phosphorylation in both cell lines at 15 min (Figure 2). However, we did not observe significant EphA2 phosphorylation after 30 min of ephrin-A5 nanocaliper stimulation except for NC-40. Similar to previous work (25), NC-40 resulted in the highest level of receptor phosphorylation in MDA-MB-231 cells at 15 min, which differed from NC-0 and NC-100 (Figure 2A and B). In U3013 cells, differences in EphA2 phosphorylation were observed in NC-100 compared with NC-0, NC-14 and NC-40 stimulation at 15 min (Figure 2C and D). These spatially-induced differences in receptor phosphorylation diminished after 30 min stimulation in both cell lines, which suggests that EphA2 receptor activation is sensitive to spatial changes at a shorter stimulation time (15 min). The differences in receptor phosphorylation caused by NC-40 and NC-100 in MDA-MB-231 and U3013, respectively, may be related to differences in EphA2 protein expression. EphA2 is highly expressed in MDA-MB-231, with a 3-fold difference as compared to U3013 (Supplementary Figure S6). We hypothesize that binding of both ephrin-A5 dimers present in NC-40 to EphA2 receptors is facilitated in cell lines with high density of receptors, such as MDA-MB-321. Conversely, in U3013 cells the low density of EphA2 receptors favors binding of NC-100 compared to NC-40.

To assess the stability of DNA origami nanostructures during cell stimulation, we investigated the integrity of ephrin-A5 nanocalipers by agarose gel electrophoresis and TEM imaging (Supplementary Figure S7). We observed that the ephrin-A5 nanocalipers remained intact after 15 and 30 min at 37°C, indicating that ligand spatial control was retained throughout cell stimulation.

### The nanoscale spatial distribution of ephrin-A5 tunes the EphA2 transcriptional response in U3013 cells

Using RNA-seq, we first investigated the transcriptional responses of EphA2 activation in the two cell lines stimulated with IgG-clustered ephrin-A5, which is a classical but spatially-uncontrolled method of EphA2 stimulation (Supplementary Figure S8 and Supplementary Table S2). There were 19 differentially expressed (DE) genes found common in MDA-MB-231 and U3013 (Supplementary Figure S8A). U3013 cells showed 24 significantly enriched pathways associated with EphA2 signaling such as MAPK signaling, lysosome and NGF signaling as well as pathways further downstream, like the cell cycle pathway (Supplementary Figure S8B). In contrast, MDA-MB-231 cells showed only five significantly enriched pathways, of which two pathways overlap with those found in U3013 cells. Within those two pathways, no DE genes were found to overlap between the two cell lines (Supplementary Figure S8C). These results indicate that U3013 cells are more transcriptionally responsive to EphA2 activation than MDA-MB-231 cells.

**Figure 3.**
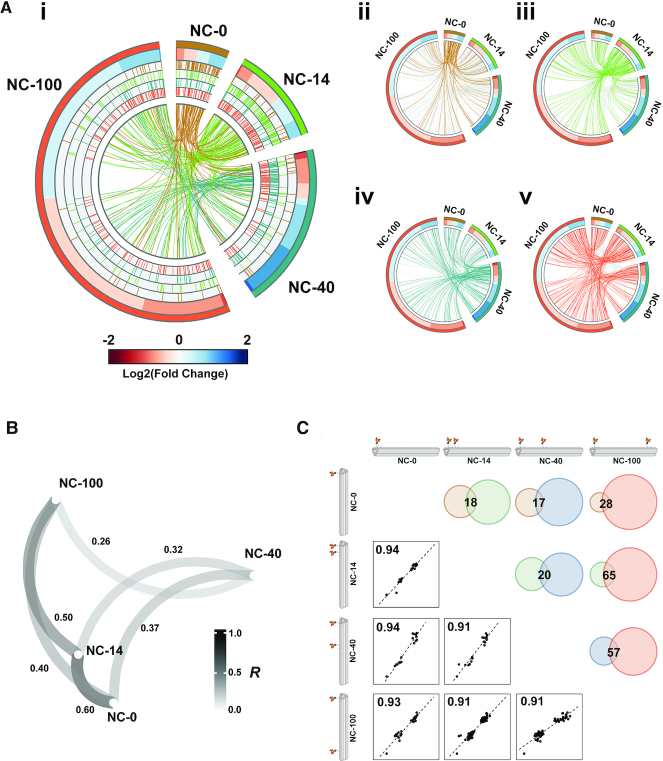
U3013 cell transcriptome upon stimulation with ephrin-A5 nanocalipers. (**A**) (i) Circos plot depicting the fold changes of DE genes in cells stimulated with NC-0 (outer rim, dark orange), NC-14 (outer rim, green), NC-40 (outer rim, teal) and NC-100 (outer rim, orange). The size of each segment reflects the number of DE genes. The inner rims show the overlap of each data set with each other, each line representing one gene. The centre core shows the overall gene overlap between all data sets as represented by links. (ii–iv) The gene overlaps of one nanocaliper data set with the other data sets. ii. NC-0, iii. NC-14, iv. NC-40 and v. NC-100. Each gene overlap is represented by a link. (**B**) Correlation analysis of the complete nanocaliper data sets. The closer the data sets are to each other, the higher their correlation is. This is also represented by the thickness and the color of the line. The value next to each line corresponds to the correlation coefficient between two data sets. (**C**) Correlation analysis of the fold changes of gene overlaps between the nanocaliper data sets. Proportional Venn diagrams show the gene overlaps between the various data sets.

Therefore, we selected U3013 cells to study the effects of the spatial distribution of ephrin-A5 on EphA2 transcriptome. We performed RNA-seq on these cells treated with ephrin-A5 nanocalipers for 30 mins (GSE138623) (Figure 3 and Supplementary Table S3). This timing was chosen to allow for the spatial effect in EphA2 activation to be translated into gene expression. The number of DE genes increased with increasing ephrin-A5 dimers spatial distance, as seen by 96, 174, 296 and 840 DE genes in NC-0, NC-14, NC-40 and NC-100, respectively (adj. *P*-value <0.05) (Figure 3A, i). On the other hand, we observed a decrease in the percentages of DE genes that overlap with one or more nanocaliper data sets with increasing ephrin-A5 spatial distance. These overlaps were 48% in NC-0, 45% in NC-14, 24% in NC-40 and 15% in NC-100 (Figure 3A, ii–iv). This divergence of EphA2-mediated transcriptional responses to increasing spacings between ephrin-A5 dimers was further supported by a correlation analysis. By comparing the fold changes of genes found common in all four nanocaliper datasets, NC-0 and the NC-14 data sets cluster relatively close together with a correlation coefficient of 0.6 (Figure 3B). NC-40 and NC-100 data sets did not cluster closely with any other data sets, indicating that they are distinct transcriptional responses. However, it should be noted that the overall correlations are relatively low between the data sets. Pairwise correlations revealed 17–65 overlapping DE genes with *R*^2^ values of above 0.91, with most genes showing highly similar fold changes (Figure 3C). We also validated the RNA-seq data by qRT-PCR and observed similar expression patterns of genes linked to the MAPK signaling, *fos* and *jun*, in both methods (Supplementary Figure S9). Overall, these results indicate a divergent EphA2 transcriptional response with a relatively modest DE gene overlap between the different ephrin-A5 nanocalipers.

### Differential pathway enrichment upon spatially-controlled EphA2 receptor activation

**Figure 4.**
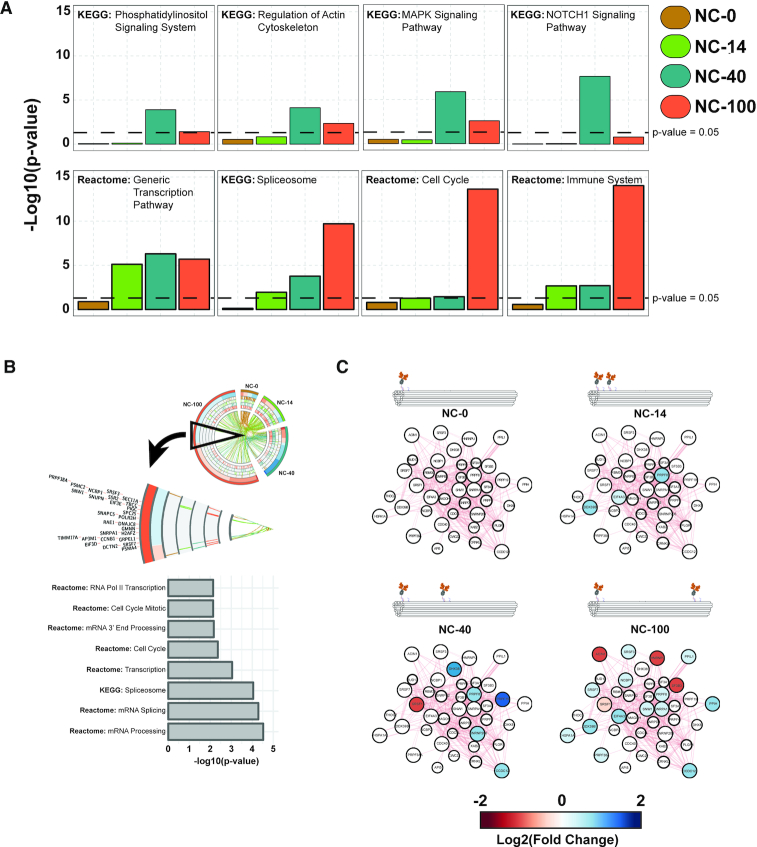
Differential significantly enriched pathways in U3013 cells stimulated with ephrin-A5 nanocalipers. (**A**) Hypergeometric probability analysis of eight selected significantly enriched pathways in at least one data set. The dotted line signifies a *P*-value of 0.05. (**B**) A subset of genes in NC-100 data set which do not overlap with other nanocaliper data sets from the GSEA. (**C**) A subset of the spliceosome network of the DE genes of each nanocaliper data set overlaid with their respective –log_2_(FC) (colored).

We further investigated the pathways in all of the nanocaliper data sets and analyzed selected pathways that are significantly enriched in one or more data sets. Stimulation with NC-40 led to activation of pathways such as phosphatidylinositol, MAPK and NOTCH1 signaling as well as actin cytoskeleton regulation (Figure 4A). On the other hand, NC-100 stimulation primarily affected the spliceosome, cell cycle and immune system pathways. These pathways either had a low significant enrichment or no enrichment at all in the other nanocaliper data sets. Interestingly, the spliceosome pathway showed an enrichment pattern that increases ‘step-wise’ as spatial distances of ephrin-A5 increase, with NC-100 being the most significant data set followed by NC-40, NC-14 and finally NC-0 being not significant. NC-0 stimulation resulted in few DE genes for any pathway to be significantly enriched. The NC-14 data set was significantly enriched for the generic transcription pathway, the immune system and the spliceosome pathways.

Interestingly, the enrichment of the spliceosome and cell cycle pathways in the NC-100 data set was found to be linked to a subset of genes with low fold changes that does not overlap with any other nanocaliper data sets. This subset accounts for ∼12.3% of the entire NC-100 data set (Figure 4B). By overlaying the DE genes of the nanocaliper data sets onto the spliceosome network, we found that the number of genes increases as the spatial distance between the ephrin-A5 dimers increases and that there was very little overlap between the different nanocaliper stimulations (Figure 4C).

As the NC-100 stimulation resulted in the most significant enrichment in cell cycle signaling, we investigated whether it could affect the cell cycle profile of U3013 cells. However, we did not detect any apparent difference in the cell cycle distribution between NC-empty and NC-100 stimulations after 24-hour incubation, indicating that at these time-scales, NC-100 stimulation does not have a major effect on cell cycle progression (Supplementary Figure S10).

**Figure 5.**
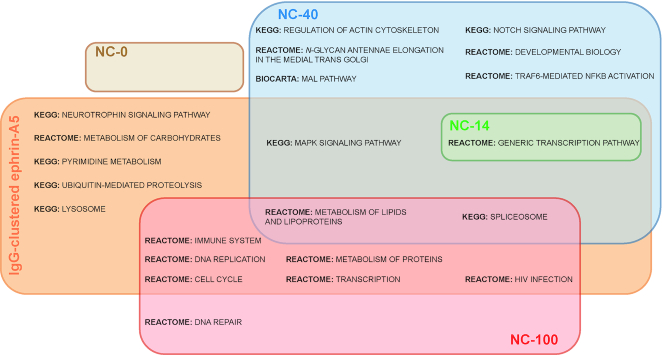
Comparison of significantly enriched pathways between spatially controlled and uncontrolled-EphA2 stimulation. Euler diagram of significantly enriched pathways of ephrin-A5 nanocaliper data sets and IgG-clustered ephrin-A5.

Finally, we compared the significantly enriched pathways activated by the ephrin-A5 nanocalipers and pathways by IgG-clustered ephrin-A5. We found that there were more overlapping pathways from NC-40 and NC-100 than from NC-0 and NC-14 stimulations (Figure 5). It is interesting to note that while NC-40 and NC-100 generated a distinct subset of pathways, they were found to be part of a larger response from IgG-clustered ephrin-A5 stimulation. Several pathways such as actin cytoskeleton regulation and NOTCH signaling were the exceptions as they were only affected by NC-40 stimulation but not by IgG-clustered ephrin-A5 stimulation. Due to the effects of IgG-clustered ephrin-A5 stimulation on cancer invasion signaling pathways, like pathothenate and CoA biosynthesis, Rho GTPases and neurotrophin signaling, we sought to investigate whether this would lead a functional outcome. However, we found that IgG-clustered ephrin-A5 stimulation did not change the invasiveness of U3013 cells as compared to IgG alone (Supplementary Figure S11).

Overall, these findings show that the ephrin-A5 nanocalipers, specifically NC-40 and NC-100, are capable of activating the EphA2 signaling transcriptome in U3013 cells in a selective manner.

## DISCUSSION

In this study, we used DNA origami nanofabrication to demonstrate that EphA2 receptor activation and downstream transcriptional responses in human glioblastoma cells are modulated by the nanoscale spatial distribution of ephrin-A5 ligands. In contrast, MDA-MB-231 cells had poor EphA2-mediated transcriptome responses despite showing differential spatially-controlled receptor activation.

We showed that the EphA2 transcriptional response becomes larger as ephrin-A5 dimers are spaced further apart from each other on the nanocalipers. Specifically, NC-100 is more efficient in triggering EphA2 downstream signaling than NC-40. The transcriptional response also becomes more divergent in terms of which pathways are being affected. Stimulation with NC-40 affects pathways linked to canonical EphA2 activation, such as phosphatidylinositol, MAPK and NOTCH1 signaling, whereas NC-100 appears to affect pathways downstream of those affected by NC-40 stimulation, such as cell cycle regulation (4,10,46). Correlation analyses performed on the complete gene expression datasets confirm this divergent nature of the two data sets. Moreover, NC-40 and NC-100 both affect distinct subsets of pathways with little overlap between them. On the other hand, NC-0 and NC-14 were found to cluster closely together, giving a similar overall response. This suggests that NC-0 and NC-14 cause similar EphA2 clustering and activation, with NC-14 providing the largest response due to the presence of two ephrin-A5 dimers rather than one on the DNA nanocaliper.

The protein-DNA nanocaliper production pipeline optimized in this study provides a framework to study spatially-controlled transcriptional responses. Spatially-uncontrolled methods for cell stimulation with multimeric ligands, like IgG-clustered ephrin-A5, result in heterogeneous higher-order structures that stimulate EphA2 in diverse ways. By tuning the spatial distance of membrane proteins with DNA nanocalipers, it is possible to specifically target subsets of downstream signaling pathways. This may be relevant when targeting only certain pathways in Eph-mediated signaling as the latter mediates both proto-oncogenic and tumor suppressor pathways (9,47).

## DATA AVAILABILITY

Raw and processed RNA-seq data sets for the reported sequencing experiments have been deposited with the Gene Expression Omnibus (GEO) under accession number GSE138620, for the IgG-clustered ephrin-A5 data set, and accession number GSE138622 for the ephrin-A5 nanocaliper data sets. Both data sets are deposited as part of the superseries GSE138623.

## Supplementary Material

gkaa274_Supplemental_Files
