# Supplementary material for: Spatial organization-dependent EphA2 transcriptional responses revealed by ligand nanocalipers
Source: Nucleic Acids Res. 2020 Apr 30;48(10):5777–87. doi: 10.1093/nar/gkaa274 (PMC7261182; doi:10.1093/nar/gkaa274)
Supplement: gkaa274_Supplemental_Files [file gkaa274_supplemental_files.zip › Supplementary_Text_revised.docx]

Supplementary Material for

**Spatial organization dependent EphA2 transcriptional responses revealed by ligand nanocalipers**

Toon Verheyen^1,†^, Trixy Fang^1,†^, Dominik Lindenhofer^1^,Yang Wang^1^, Karen Akopyan^2^, Arne Lindqvist^2^ , Björn Högberg^1^ and  Ana I Teixeira^1*^

Supplementary Methods

Reducing SDS-PAGE and protein staining

Two micrograms of purified ephrin-A5-oligo conjugates and unconjugated ephrin-A5-Fc were mixed with 1× NuPAGE reducing agent and 1× NuPAGE LDS sample buffer and heated at 70°C for 10 min. The mixes were ran on a 4-12% NuPAGE Bis-Tris gel in 1× NuPAGE MES SDS running buffer for 35 min at 200V. The gel was then stained with SimplyBlue Safe Stain and destained according to manufacturer’s instructions.

Nanocaliper gel migration analysis

For each replicate, line plots were drawn across all the lanes based on the DNA ladder. The lowest value obtained from all the line plots on the gel was used for background subtraction. The maximum grey value in each lane was assumed to be the highest peak and the migration distance, as given according to the line plot, was determined. The migration distance was normalized by setting the maximum grey value of NC-empty as the midpoint to normalize the migration distances of the other nanocalipers. The normalized data was combined and then plotted in *R* using the *ggplot2* package (v. 3.2.0)(1).

*In vitro* caliper binding assay

The ephrin-A5 nanocalipers were pre-incubated with recombinant human EphA2 protein (R&D Systems) at a molar ratio of 1:10 for 1 h at room temperature. The mix was then run on a 1.5% agarose-TBE gel supplemented with 10 mM MgCl_2_. The gel was run in 1× TBE + 13 mM MgCl_2_ for 2-3 h in an ice-water container at 90 V and imaged with UV transillumination. The gel image was analyzed using ImageJ (v. 1.52a).

SDS-PAGE and Western blot

Whole cell lysates of 1×10^6^ MDA-MB-231 and U3013 cells were prepared using RIPA Lysis and Extraction Buffer supplemented with 1×Halt Protease Inhibitor Cocktail (ThermoFisher Scientific) according to manufacturer’s instructions. The lysates were mixed with 1× NuPAGE LDS Sample Buffer (ThermoFisher Scientific), boiled at 70°C for 2 min and loaded onto NuPAGE Novex 4-12% Bis-Tris gels with 25 µg total protein in each well. The lysates were run at 200 V in 1× NuPAGE MOPS SDS Running Buffer (ThermoFisher Scientific) for 55 min. Proteins were transferred onto nitrocellulose membranes of 0.2 µm pore size (ThermoFisher Scientific) in Towbin buffer (25 mM Tris, 190 mM glycine, 20% methanol (v/v), 10% SDS (v/v), pH 8.3), 30 V for 2 h at 4°C. To visualize total protein content, membranes were stained with 1% (w/v) Ponceau S (Sigma-Aldrich) in 5% acetic acid for 10 min. Membranes were then destained several times with 1×TBS with 0.1% Tween-20 (TBST) and blocked with 3% BSA in TBST for 1 h at room temperature.  The membranes were incubated with rabbit anti-EphA2 antibodies (Cell Signalling Technology, #6997) (1:500 dilution in TBST with 1% BSA) overnight at 4°C. After overnight incubation and washing with TBST washes, membranes were incubated with horseradish peroxidase-conjugated goat anti-rabbit secondary IgG (ThermoFisher Scientific, #32460) (1:3000 dilution in TBST with 1% BSA) for 1 h at room temperature. Protein signals were detected using Pierce ECL Plus Western Blotting Substrate (ThermoFisher Scientific) and imaged with ImageQuant LAS 4010 system (GE Healthcare).  The resulting blots were quantified using ImageJ to calculate EphA2 protein level of MDA-MB-231 cells relative to U3013 cells. Grey values obtained from the EphA2 band were normalized over the grey values from total protein content based on the Ponceau stain. The normalized EphA2 band originating from MDA-MB-231 lane was then normalized over the normalized value of the U3013 band, which was then set to 1.

Negative-stain TEM

An aliquot of the various ephrin-A5 nanocalipers (NC-empty, NC-0, NC-14, NC-40 and NC-100) were incubated either at 37° C or at room temperature for 30 min. A 5-μL aliquot of 3 nM DNA nanocaliper structure sample was spotted on a glow-discharged, carbon-coated, formvar resin grid (Electron Microscopy Sciences) for 40 seconds before blotting on a filter paper, and then stained with fresh 2% w/v uranyl formate solution in deuterium oxide for 40 seconds. The stained sample was imaged using a Talos 120C G2 with Ceta-D detector transmission electron microscope at 120 kV with magnifications 73,000.

jPLA plugin

The full plugin workflow is shown in Supplementary Figure S5. In brief, raw microscope images, i.e. “.czi” or “.zvi”, containing single cells were used as input. The cell (phalliodin) or nucleus (DAPI) size was first determined in order for cell recognition to be successful later on. Once this was determined, the plugin was run and channels for the cell, nuclei and PLA signals as well as the feature size and z-stack range were specified. The plugin will then create a maximum intensity projection based on the z-stack range given. The PLA channel was inverted and subjected to background subtraction using a rolling ball radius of 10. The cell or nuclei channel was then subjected to background subtraction using a background creation and subsequent image subtraction method. A mask was created from the cell or nuclei channel and overlaid on top of the PLA signal. The PLA signal within the mask outline was counted using the “Count Maxima” function in ImageJ. Once the analysis has finished, a tab-delimited text file is provided as output containing the raw PLA dot count per cell. This was then directly read into R to further analyze the data.

qRT-PCR

U3013 cells were plated at 4,000 cells per well in a 96-well plate and allowed to attach for 24 hrs. After attachment, the cells were either stimulated with 50 µl of ephrin-A5 nanocalipers, 20 µg/ml AffiniPure Goat Anti-Human IgG, Fcγ Fragment Specific (Jackson Immunoresearch, 109-005-008) or IgG-clustered ephrin-A5 (pre-clustered with IgG at a mass ratio of 1:10 for 15 min at room temperature) for 30 min. The cells were then processed using the Power SYBR Green Cells-to-C_t_kit (ThermoFisher Scientific) following manufacturer’s guidelines. qRT-PCR was performed to study expression of genes, FOS (Fw: 5’ GCCGGGGATAGCCTCTCTT 3’, Rev: 5’ GCAGTGACCGTGGGAATGA 3’) and JUN (Fw: 5’ GAGAGGAAGCGCATGAGAA 3’, Rev: 5’ TGCAACTGCTGCGTTAGCAT 3’). The primers for the reference gene ACTIN, were obtained from the SYBR Green Cells-to-C_T_control kit (ThermoFisher Scientific). Expression fold changes were determined using the Pfaffl method as previously described with the primer efficiencies set to a value of 2 (2).

Cell cycle experiment (EdU labeling)

U3013 cells were seeded on a poly-ornithine and laminin-coated 96-well plate at 2000 cells per well for 24 h before stimulation with 20 nM NC-empty, NC-0 or 10 nM NC-100 in 1×PBS with 13 mM MgCl_2_, 10 ng/ml EGF and FGF for 30 min at 37°C, 5% CO_2_. After stimulation, fresh GBM media was added and cells were incubated for 24 h at 37°C, 5% CO_2_.  For cell cycle progression measurements, U3013 cells were treated with 10mM EdU (5-ethynyl-2′-deoxyuridine, Molecular Probes) for 1 h before fixation. Fixed samples were washed in DPBS (Gibco) and incubated with DAPI for 1 h at room temperature. EdU-click chemistry was performed by incubation in 100mM Tris, 1mM CuSO_4_, 100mM ascorbic acid and fluorescent dye azide for 30 min at room temperature. Images were captured on Leica DMI6000 Imaging System with 20 (NA 0.4) air objective and analyzed by CellProfiler (3). Two independent experiments were performed, from two different cell passages, with a total of four technical replicates.

Cell invasion assay

The assay was performed using QCM ECMatrix 24-well cell invasion assay with fluorimetric detection (Merck Milipore). To rehydrate the extracellular matrix membrane, each well insert was kept in GBM media without growth factors and antibiotics for 2 h at room temperature before cell seeding. U3013 cells were stimulated with 20 µg/ml AffiniPure Goat Anti-Human IgG, Fcγ Fragment Specific (Jackson Immunoresearch, 109-005-008) or IgG-clustered ephrin-A5 (pre-clustered with IgG at a mass ratio of 1:10 for 15 min at room temperature) for 30 min at 37°C, 5% CO_2_. 40,000 cells were seeded into each insert in 200 µl GBM media without growth factors and antibiotics and the lower chambers were filled with 500 µl GBM media with growth factors. Cells were incubated for 48 h at 37°C, 5% CO_2_ and then lysed for CyQuant fluorescence measurement at 520 nm following assay’s instructions.

Supplementary References

1. Wilkinson,L. (2011) ggplot2: Elegant Graphics for Data Analysis by WICKHAM, H. *Biometrics*, 10.1111/j.1541-0420.2011.01616.x.

2. Pfaffl,M.W. (2001) A new mathematical model for relative quantification in real-time RT-PCR. *Nucleic Acids Res.*, **29**, e45.

3. McQuin,C., Goodman,A., Chernyshev,V., Kamentsky,L., Cimini,B.A., Karhohs,K.W., Doan,M., Ding,L., Rafelski,S.M., Thirstrup,D., *et al.* (2018) CellProfiler 3.0: Next-generation image processing for biology. *PLoS Biol.*, 10.1371/journal.pbio.2005970.

Supplementary Table Legends

**Supplementary Table S1. Nanocaliper Staples.** This lists all the “staple” oligos required to produce the DNA nanocalipers described in the paper. The excel file consists of 3 sheets, “Core1”, “Core2” and “ProtrudingStaples”. Each table has three columns, “WellPostion” refers to the position of the oligo in a 96-well plate format, “Name” refers to the name of the oligo and “Sequence” is the oligo sequence. The two plates labelled “Core” are necessary in all DNA nanocaliper structures, the plate “ProtrudingStaples” will need to be handles as described in Figure S1.

**Supplementary Table S2. IgG-clustered RNA-seq Data.** This excel file lists all the differentially expressed (DE) genes detected in both cell lines, MDA-MB-231 and U3013, upon stimulation with IgG- clustered ephrin-A5. The excel file is split up into 2 sheets, containing either the DE genes of the MDA- MB-231 or U3013 data sets and is the raw output of the DESEQ2 package.

**Supplementary Table S3. Nanocaliper RNA-seq Data.** This excel file lists all the differentially expressed (DE) genes detected in all 4 ephrin-A5 nanocaliper data sets. The excel file is split up into 4 sheets, containing either the DE genes of the NC-0, NC-14, NC-40 or NC-100 data sets and is the raw output of the DESEQ2 package.
