# Supplementary material for: Spatial organization-dependent EphA2 transcriptional responses revealed by ligand nanocalipers
Source: Nucleic Acids Res. 2020 Apr 30;48(10):5777–87. doi: 10.1093/nar/gkaa274 (PMC7261182; doi:10.1093/nar/gkaa274)
Supplement: gkaa274_Supplemental_Files [file gkaa274_supplemental_files.zip › Verheyen_Fang_Supplementary_Figures_revised -.pdf]

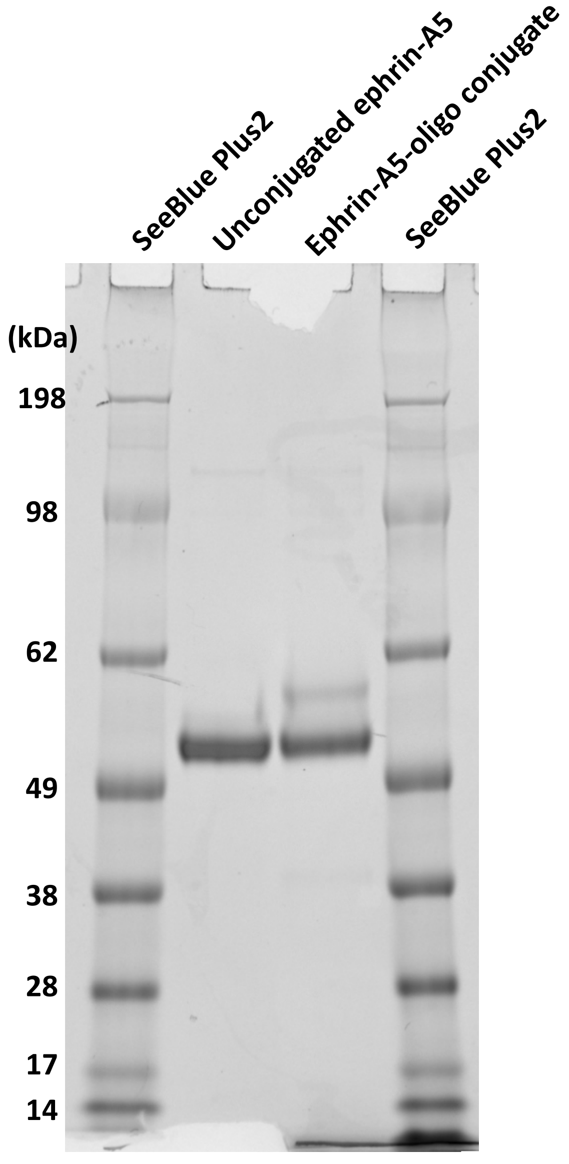

Figure S1. Analysis of ephrin-A5-oligo conjugates by 4-12% reducing SDS-PAGE.

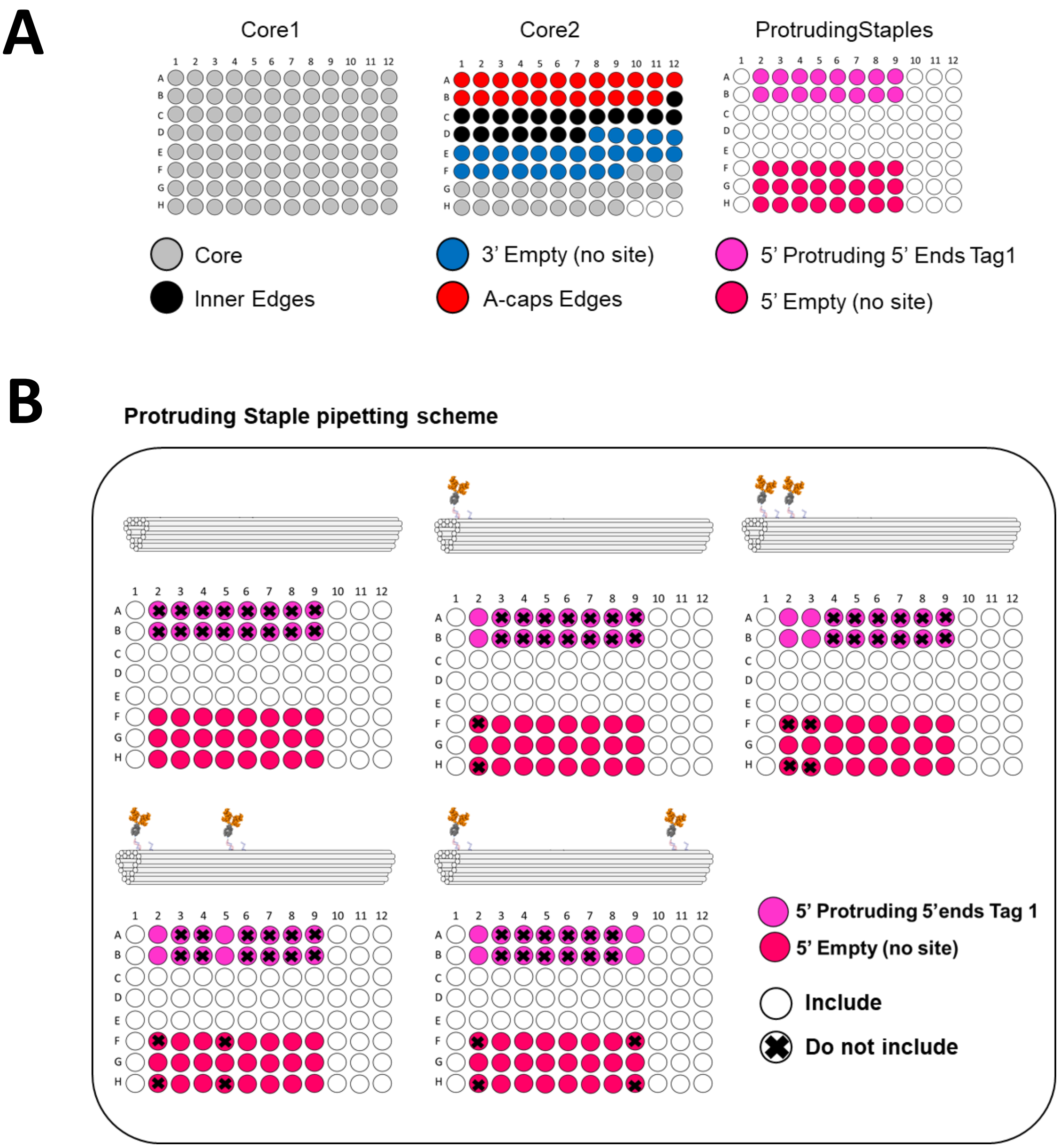

Figure S2. Staple Mix Plate Layout. The general "staple" oligo mix plate layout used to produce the DNA nanocalipers. **A.** Three plates containing the "staple" oligos needed to create DNA nanocalipers. The "Core 1" and "Core 2" plates represent the oligos that should always be included in the "staple" pool. The "ProtrudingStaples" plate represent the oligos that are either protruding from the DNA nanocalipers or are the non-protruding equivalents. These should be added based on the type of DNA nanocaliper produced. **B.** The pipetting scheme for the protruding staples. Based on the DNA nanocaliper being produced, a different combination of protruding and non-protruding strands need to be added to the "staple" pool. Coloured wells indicated with a cross should not be added whereas coloured wells without a cross should be added. The sequences and the plates are described in Supplementary Table S1.

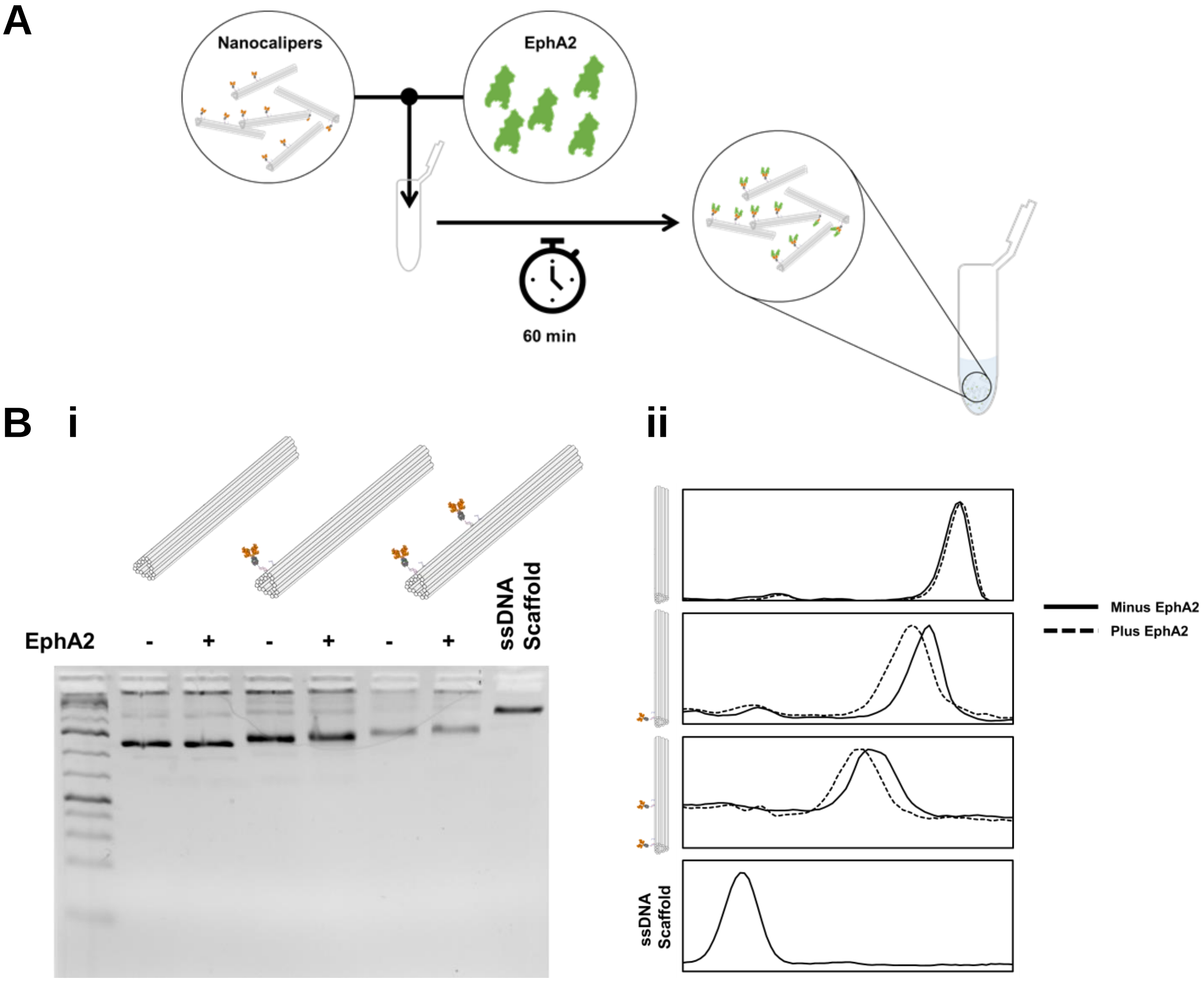

Figure S3. Gel shift binding assay. This assay is designed to test the ability of ephrin-A5 to interact with a recombinant extracellular domain of the EphA2 receptor. **A.** A schematic showing the principle of the binding assay. **B. i.** A 1.5% agarose gel showing the DNA nanocalipers with or without EphA2 bound to the ephrin-A5 dimers hybridized to the DNA nanocalipers. **ii.** Line plots drawn across the gel bands were overlaid on top of each other to indicate the band shift observed within each type of DNA nanocaliper.

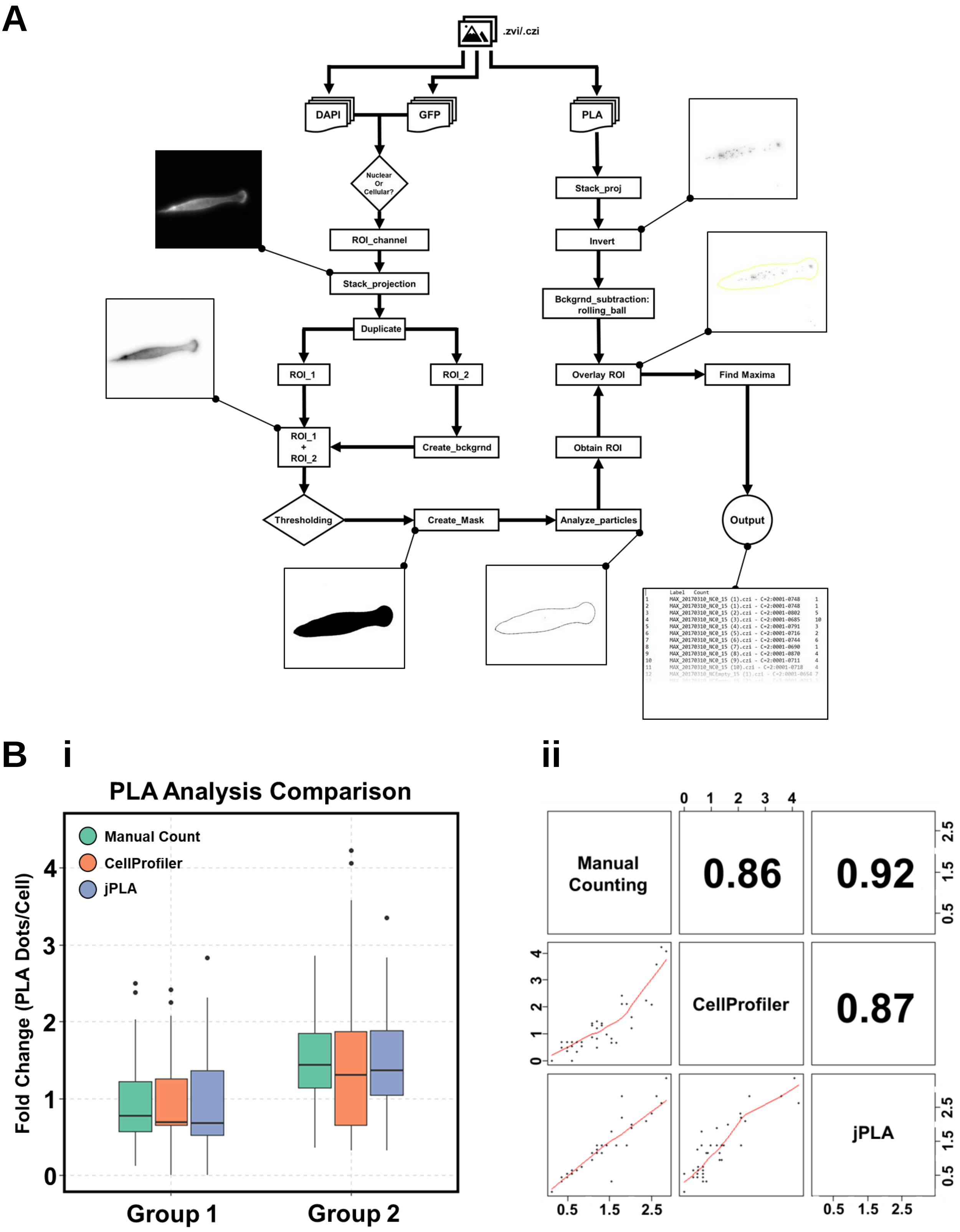

Figure S4. jPLA: a simple PLA Analysis tool. A. Diagram showing the workflow of the jPLA macro. **B.** A comparison between three methods of quantification, Manual count, CellProfiler and jPLA. **i.** A box plot showing the analysis of two groups of 20 (Group 1) and 19 (Group 2) microscope images by the three different analysis methods. **ii.** Correlation analysis performed comparing all three methods.

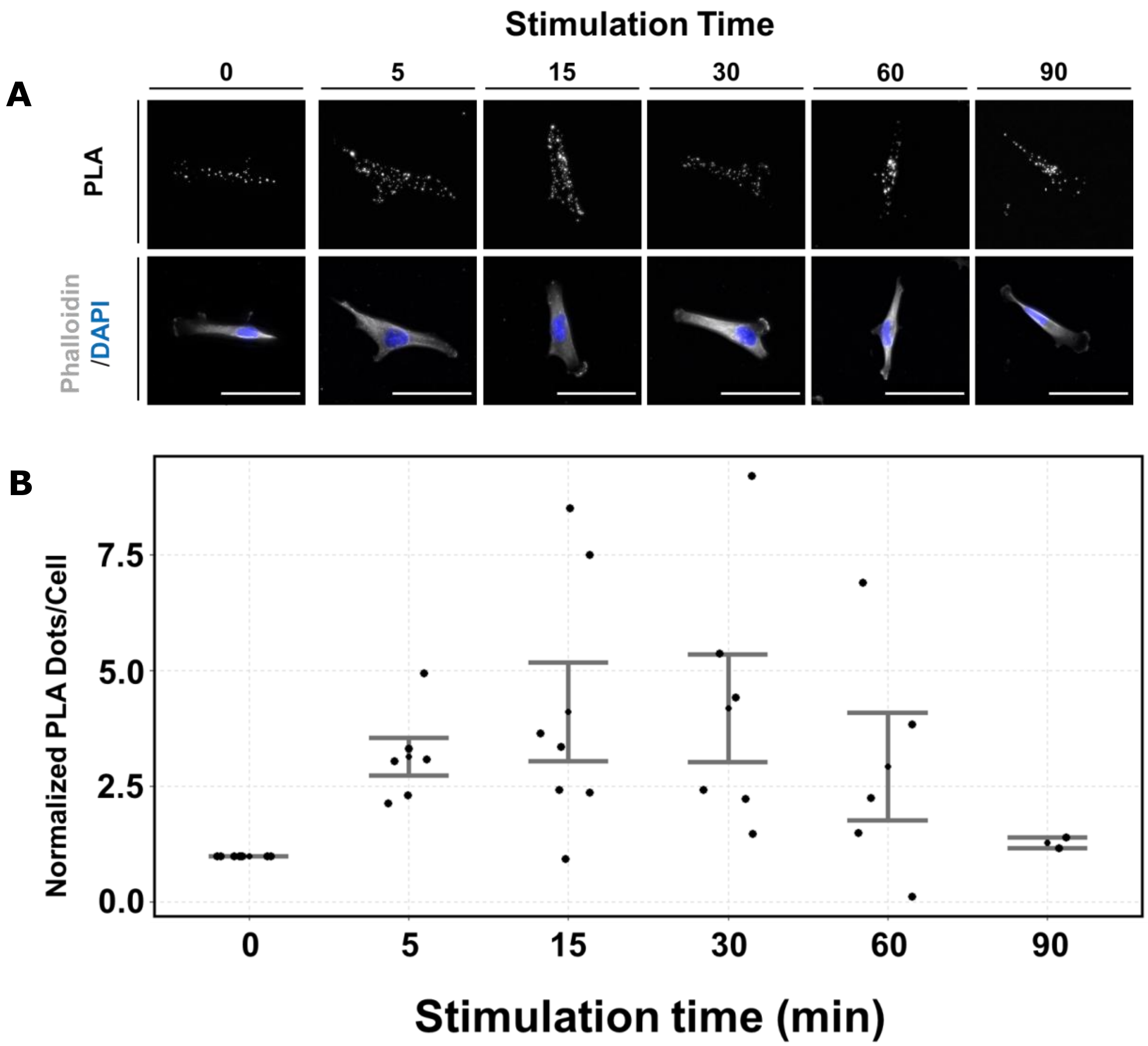

Figure S5. Time-series of EphA2 receptor activation by IgG-clustered ephrin-A5 stimulation of EphA2 in MDA-MB-231 cell line determined by PLA. A. Representative microscope images of the PLA signal observed at different timepoints of stimulation. The images show the pTyr/EphA2 PLA signal and a merged images showing both phalloidin staining (white) and DAPI (blue). Scale bar denotes 50 µm. **B.** Plot of the normalized PLA signal per cell over time. Each dot represents one independent experiment of 10-20 cells analyzed. Error bars indicate the standard error of the mean.

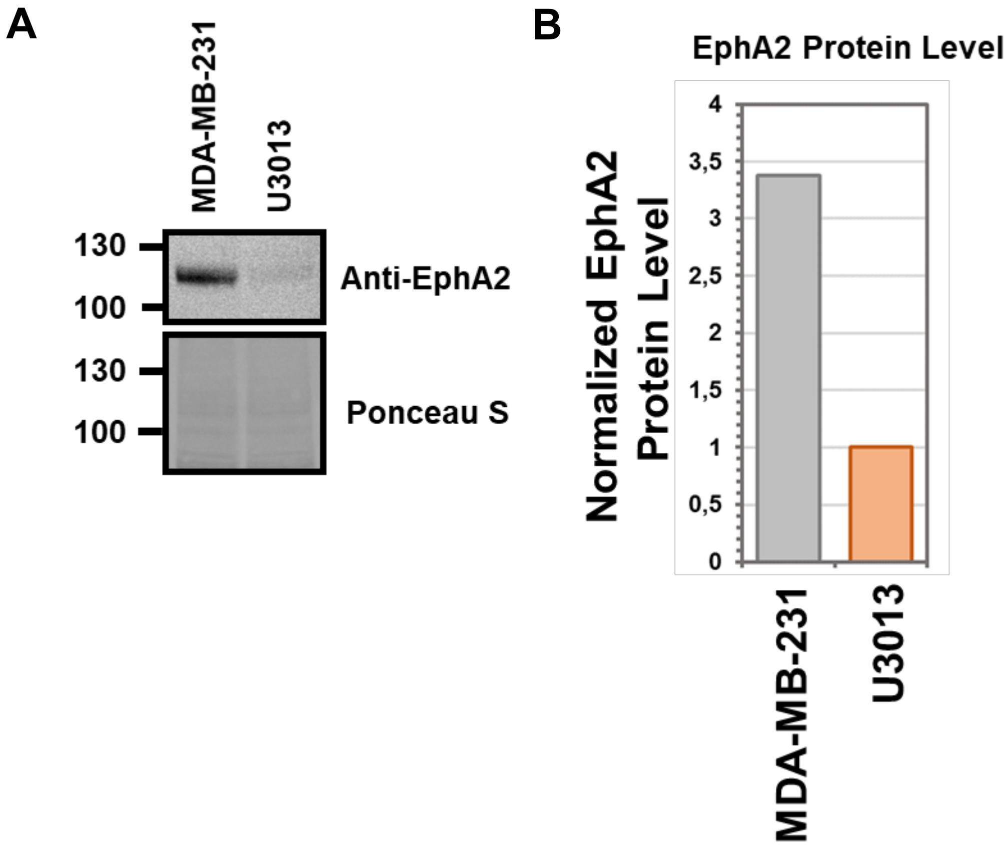

Figure S6. EphA2 protein level in MDA-MB231 and U3013 cell lines. A. Western blot of protein lysates obtained from MDA-MB-231 and U3013 cell lines. Ponceau S staining was included as a loading control. **B.** Normalized EphA2 protein levels based on the Ponceau S staining in both cell lines.

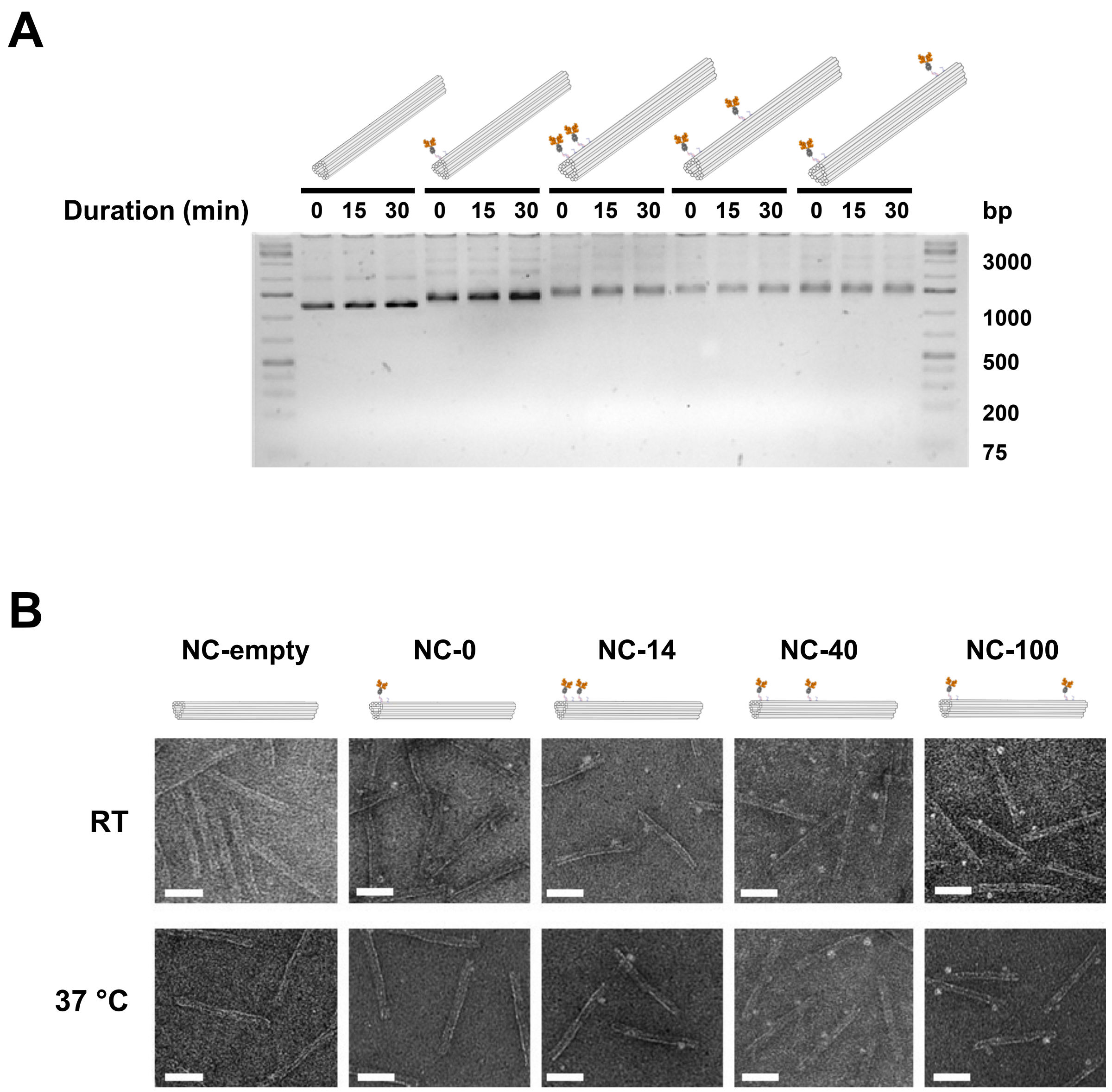

Figure S7. Stability of ephrin-A5 nanocalipers. A. Agarose gel showing the stability of 5 different ephrin-A5 nanocaliper designs before or after 15 and 30 min of cell stimulation. **B.** TEM images showing ephrin-A5 nanocalipers at room temperature and after 30 min incubation at 37°C. Scale bar denotes 50 nm.

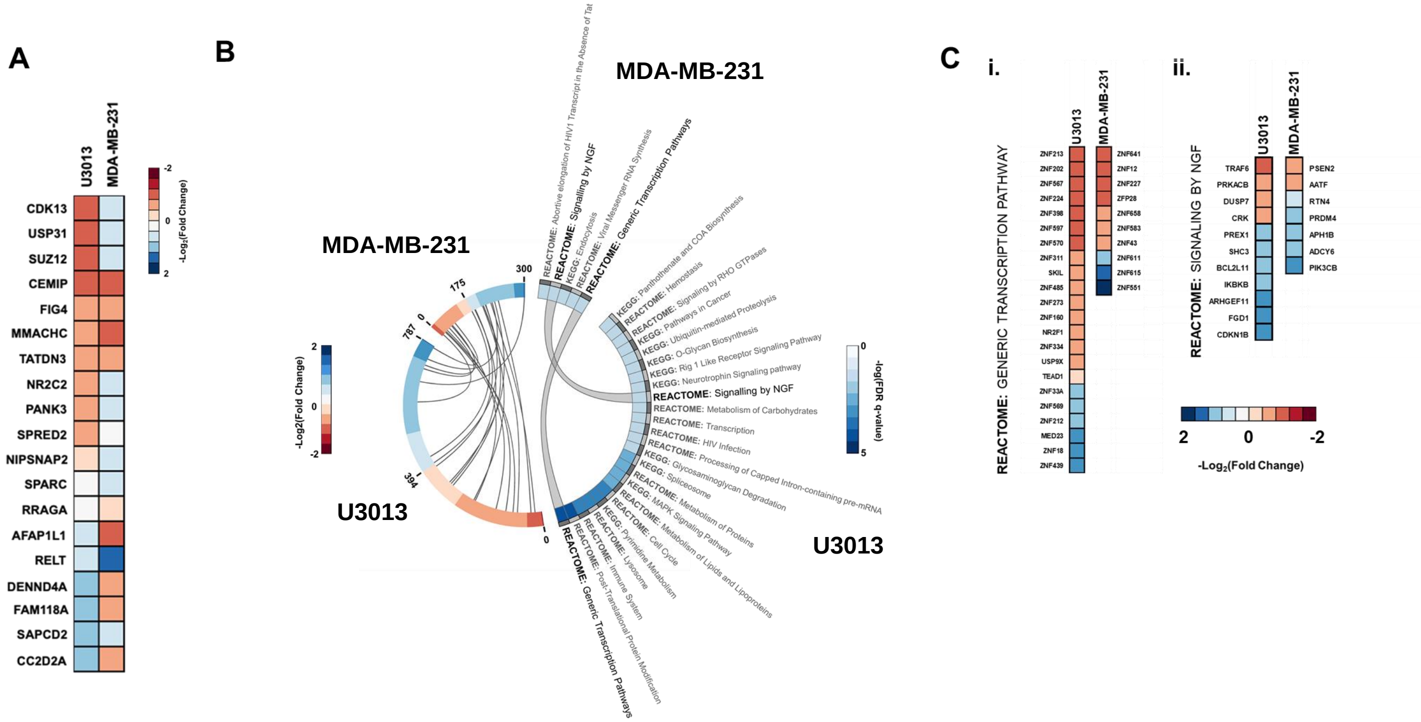

Figure S8. Transcriptome of MDA-MB-231 and U3013 cells upon stimulation with IgG-clustered ephrin-A5. A. The log2 fold changes of the DE genes in common between the two cell lines. **B**. Circos plot showing the DE log2 fold change of the DE genes associated with each cell line and the overlaps between the two datasets indicated by the black links (left). The numbers flanking the log2 fold changes indicate the number of genes. On the right hand side, the significantly enriched pathways associated with each cell line and the overlaps are indicated by shaded grey links and in bold text. **C.** Fold changes of genes significantly enriched in the data sets queried for the i. Generic Transcription Pathway and ii. Signaling by NGF. Fold changes are shown for both data sets in direct comparison. The full data set can be found under GEO accession number GSE138620 and the list of DE genes are listed in Supplementary Table S3.

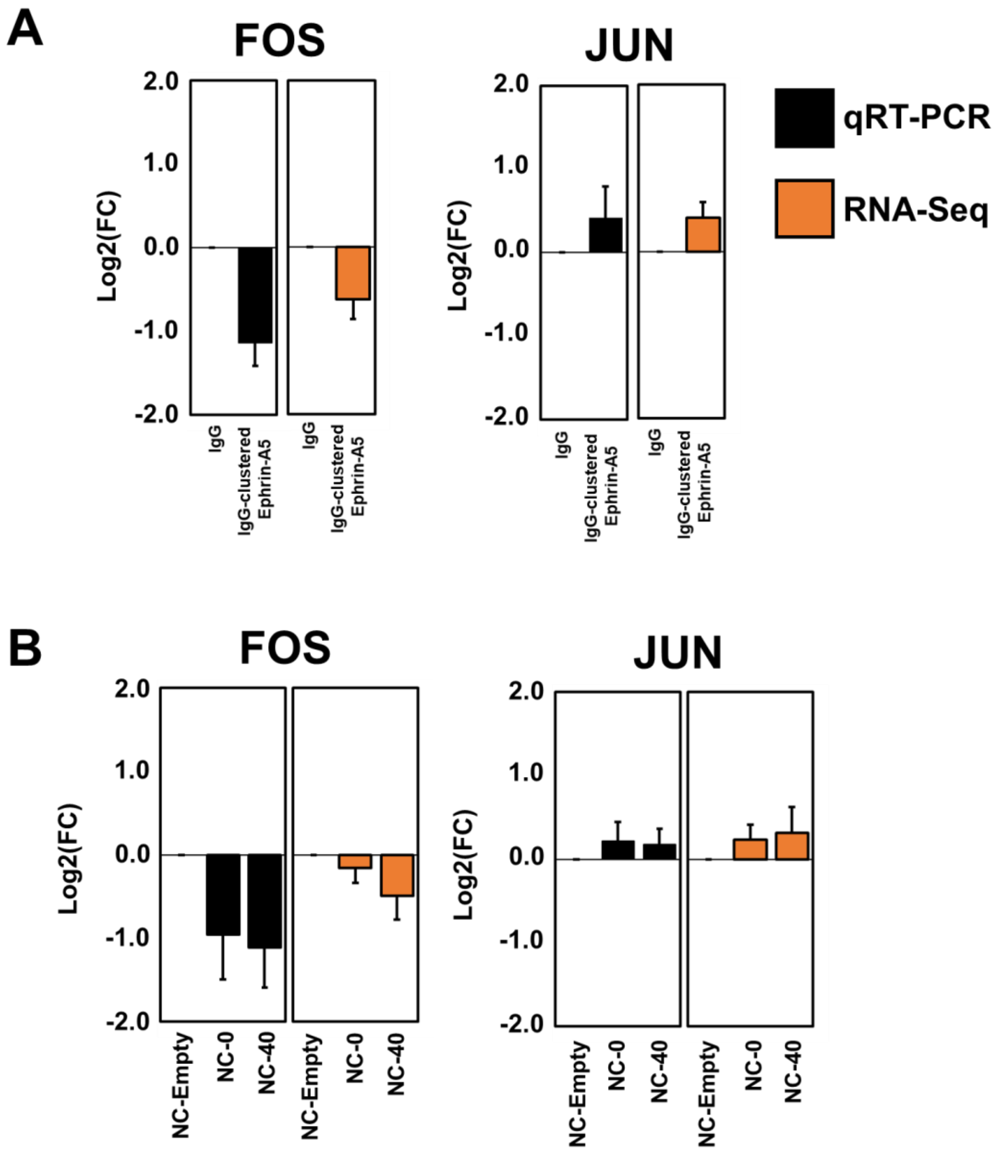

Figure S9. qRT-PCR validation of RNA-seq of the U3013 cell line. Gene Expression data of FOS and JUN of both qRT-PCR and RNA-Seq experiments on U3013 cells upon stimulation with **A.** IgG or IgG-clustered ephrin-A5 or **B.** ephrin-A5 Nanocalipers. Error bars represent standard error of log fold change.

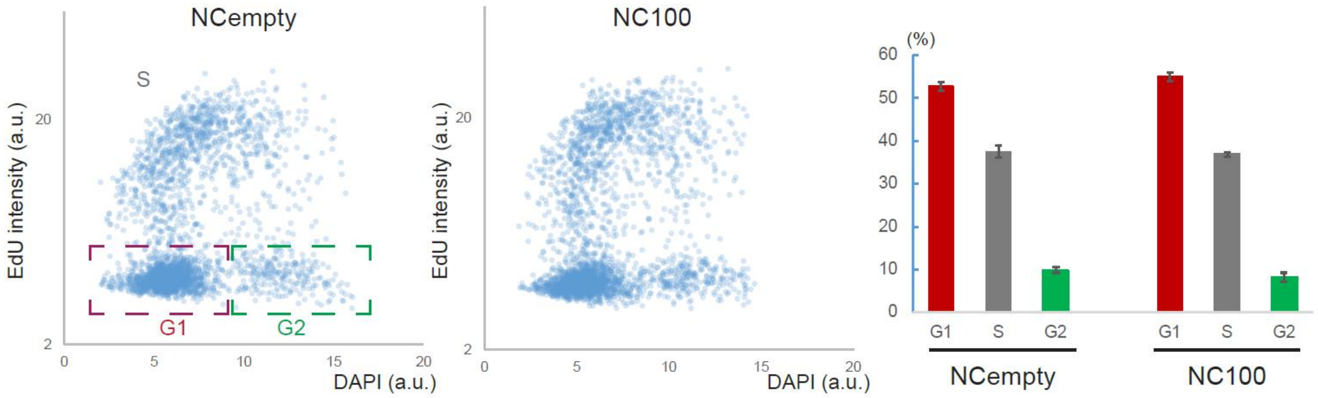

Figure S10. Cell cycle profiles of the U3013 cell line stimulated with NC-empty and NC-100. No apparent difference in cell cycle profile after treatment with NC100. U3013 cells were treated overnight with 10 nM of NC-empty or NC-100 as indicated. After a 1-hour EdU pulse, the proportion of cells in G1, S, or G2/M phase were quantified as indicated. Quantification shows average and standard deviation from two independent experiments with a total of four technical replicates.

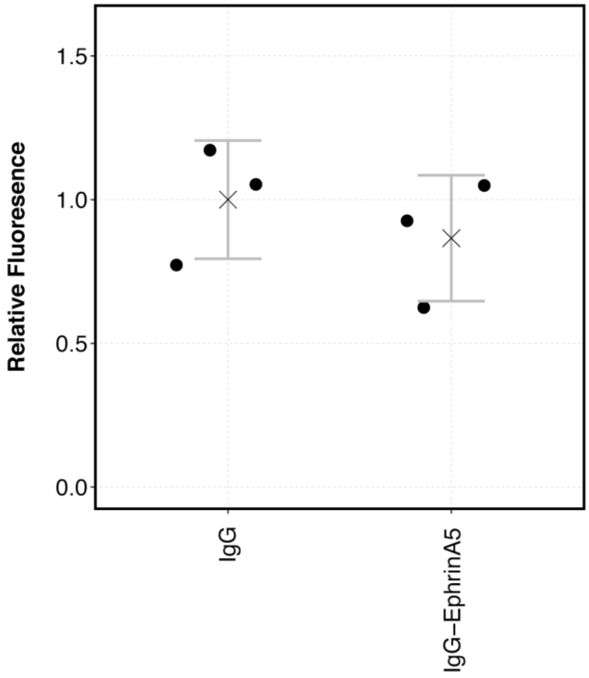

Figure S11. Effect of IgG clustered ephrin-A5 stimulation on cell invasion. Relative fluorescence from extracellular matrix invasion of U3013 cells 48 hours after stimulation with IgG or IgG-clustered ephrinA5; n = 1. Data shows mean (cross) one standard deviation (error bar) of three technical replicates.
